# High-Risk HLA-DQ Mismatches Are Associated With Adverse Outcomes After Lung Transplantation

**DOI:** 10.3389/ti.2024.13010

**Published:** 2024-09-19

**Authors:** Lisa Kleid, Julia Walter, Patrick Moehnle, Christian Wichmann, Julia Kovács, Andreas Humpe, Christian Schneider, Sebastian Michel, Nikolaus Kneidinger, Michael Irlbeck, Jan Fertmann, Andrea Dick, Teresa Kauke

**Affiliations:** ^1^ Division of Transfusion Medicine, Cell Therapeutics and Haemostaseology, University Hospital, LMU Munich, Munich, Germany; ^2^ Division of Thoracic Surgery, University Hospital, LMU Munich, Munich, Germany; ^3^ Comprehensive Pneumology Center (CPC), Helmholtz Munich, Member of the German Center of Lung Research (DZL), LMU Munich, Munich, Germany; ^4^ Department of Medicine V, LMU Munich University Hospital, Munich, Germany; ^5^ Department of Cardiac Surgery, University Hospital, LMU Munich, Munich, Germany; ^6^ Division of Pulmonology, Department of Internal Medicine, Medical University of Graz, Graz, Austria; ^7^ Department of Anaesthesiology, University Hospital, LMU Munich, Munich, Germany; ^8^ Transplantation Center Munich, LMU Munich University Hospital, Munich, Germany

**Keywords:** eplet matching, *de novo* donor specific antibody, HLA-DQ antibody, risk-stratification, lung transplantation

## Abstract

Human leukocyte antigen (HLA) mismatches (MM) between donor and recipient lead to eplet MM (epMM) in lung transplantation (LTX), which can induce the development of de-novo donor-specific HLA-antibodies (dnDSA), particularly HLA-DQ-dnDSA. Aim of our study was to identify risk factors for HLA-DQ-dnDSA development. We included all patients undergoing LTX between 2012 and 2020. All recipients/donors were typed for HLA 11-loci. Development of dnDSA was monitored 1-year post-LTX. EpMM were calculated using HLAMatchmaker. Differences in proportions and means were compared using Chi2-test and Students’ t-test. We used Kaplan-Meier curves with LogRank test and multivariate Cox regression to compare acute cellular rejection (ACR), chronic lung allograft dysfunction (CLAD) and survival. Out of 183 patients, 22.9% patients developed HLA-DQ-dnDSA. HLA-DQ-homozygous patients were more likely to develop HLA-DQ-dnDSA than HLA-DQ-heterozygous patients (*p* = 0.03). Patients homozygous for HLA-DQ1 appeared to have a higher risk of developing HLA-DQ-dnDSA if they received a donor with HLA-DQB1*03:01. Several DQ-eplets were significantly associated with HLA-DQ-dnDSA development. In the multivariate analysis HLA-DQ-dnDSA was significantly associated with ACR (*p* = 0.03) and CLAD (*p* = 0.01). HLA-DQ-homozygosity, several high-risk DQ combinations and high-risk epMM result in a higher risk for HLA-DQ-dnDSA development which negatively impact clinical outcomes. Implementation in clinical practice could improve immunological compatibility and graft outcomes.

## Graphical Abstract

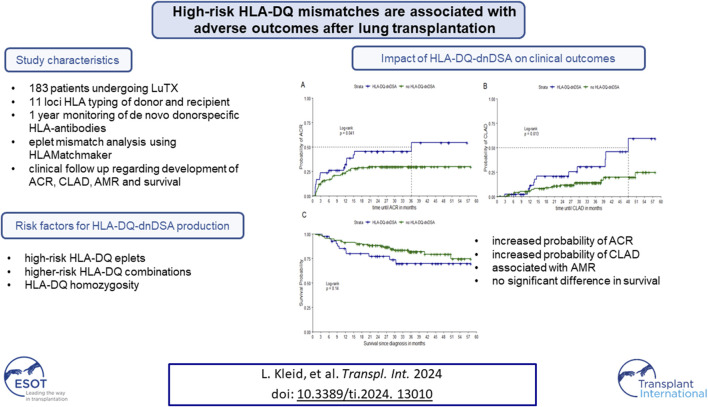

## Introduction

Lung transplantation can lead to better quality of life and prolonged survival in patients suffering from end-stage lung disease. Despite improvement of surgical techniques and advances in immunosuppression, the median survival time after lung transplantation remains at 6 years [1]. One limiting factor is the development of *de novo* donor specific HLA antibodies (dnDSA), which are part of antibody-mediated rejection (AMR) and have been associated with the development of acute and chronic rejection, primarily the bronchiolitis obliterans syndrome (BOS) [2, 3]. However, human leukocyte antigen (HLA) compatibility of donor and recipient can improve long-term graft survival of transplanted organs. Choosing histocompatible donors might reduce the risk of HLA antibody development and therefore lower the risk of rejection of the donor organ [4]. Currently, HLA-matching is not taken into account for allocation of lungs due to controversial data, urgency and organ shortage. HLA-matching is only mandatory in kidney patients and current findings show that disparities between HLA molecules are better described by epitope matching algorithms rather than matching the entire antigen [5–7]. With HLAMatchmaker, the immunogenic parts of each HLA molecule, the so-called eplets, can be calculated. Eplets are known as variable amino acid segments within a 3.0–3.5 Ångstroms radius of functional HLA epitopes, which can be directly recognized by recipients’ B-lymphocytes and thus lead to the development of donor-specific antibodies (DSA) [8, 9]. Although there is consensus among experts that molecular histocompatibility is better described by eplet mismatches than antigen mismatches, the immunogenicity of the individual eplet mismatches (epMM) is still a matter of debate [10]. There is a strong need to define immunogenicity of the eplets in order to implement epitope matching into routine diagnostics. Institutions such as the International HLA & Immunogenetics Workshop Foundation work to improve patient care by facilitating collaborations between researchers. In a recent study a German research group was able to show that immunisation against HLA-class II and especially against HLA-DQ made up the largest part of *de novo* donor-specific HLA-antibodies in their lung transplant patients [11]. Both matching algorithms, PIRCHE-II and HLAMatchmaker, have proven to be helpful tools to identify patients at higher risk for the development of *de novo* DSA, especially when used together. However, immunisation seems to be determined not only by the amount of eplets present, but also by the presence of high-risk eplets resulting from certain donor-recipient constellations [12]. The aim of our present study was to identify those eplet disparities between recipient and donor that could be associated with the development of HLA-DQ-dnDSA. Beside identifying high-risk eplets, we aimed to reveal other risk factors that are associated with HLA-DQ-dnDSA development. We also aimed to confirm the association between the development of HLA-DQ-dnDSA and acute cellular rejection (ACR), antibody-mediated rejection (AMR), chronic lung allograft dysfunction (CLAD) and survival.

Thus, we would like to present an approach to improve risk assessment in lung transplant patients to potentially improve long-term transplant outcomes.

## Patients and Methods

### Lung Transplant Cohort

This retrospective study is based on data from patients who underwent lung transplantation at the Ludwig-Maximilians-University (LMU) hospital between 2012 and 2020. The main inclusion criteria was complete HLA 11-loci loci typing of both donor and recipient (HLA-A, B, C, DRB1, DRB345, DQA1, DQB1, DPA1 and DPB1). Patients with pre-transplant HLA antibodies were excluded from the study, as were patients who did not have class I or class II HLA mismatches. The majority of the patients received a standard triple immunosuppressive regimen with tacrolimus, mycophenolat-mofetil and steroids without induction therapy. All patients were followed up at the transplant centre including lung function tests, bronchoscopy and HLA antibody screening.

Our study was approved by the ethics committee of LMU (reference number 22-0166) and was carried out in accordance with the Declaration of Helsinki, Good Clinical Practice guidelines, and local ethical and legal requirements.

### HLA Typing

All patients and donors in the study were routinely typed for 11 HLA loci. Recipients were typed by means of sequence specific oligonucleotide technique (LABType™ SSO Typing Kits, One Lambda, Inc., Canoga Park, CA, United States). Organ donors were typed using the sequence specific oligonucleotide technique (LABType™ SSO Typing Kits, One Lambda, Inc., Canoga Park, CA, United States) or real-time PCR genotyping with sequence-specific primers (LinkSēq™ HLA-ABCDRDQB1 384 Kit, One Lambda, Inc., Canoga Park, CA, United States).

### HLA-Antibody Detection

According to the local transplant protocol, patients’ sera are regularly tested for the presence of HLA antibodies using Luminex screening and single antigen bead technology prior to lung transplantation as well as 1, 3, 6 and 12 months after transplantation (LABScreen™ Mixed Class I and II and LABScreen™ Single-antigen HLA Class I - Combi and Class II – Group 1, One Lambda, Inc., Canoga Park, CA, United States). Patients’ sera have been heat-inactivated to avoid prozone effect and were measured undiluted. All donor specificities reported could be explained by one or more of the mismatched eplets. Specificities with a mean fluorescence intensity of approximately 1.000 were considered positive. The majority of dnDSA have been detected more than once and have been classified as persistent. They have been defined as transient if they disappeared spontaneously or after treatment. All of our analysis were performed with persistent and transient dnDSA.

### Antigen and Eplet Matching

HLA antigen matching was performed by comparing HLA of donor and recipient on the antigen level. In case of ambiguities, the most common alleles and their resulting serological equivalents were used. Due to the small number of patients in each group, patients and donors, homozygous either for HLA-DQ5 and/or -DQ6, were combined as HLA-DQ1 homozygous, patients homozygous for either HLA-DQ7, -DQ8 and/or DQ9 were termed as HLA-DQ3 homozygous.

Number and type of epMMs was calculated with R. Duquesnoy’s HLAMatchmaker algorithm (HLAMatchmaker algorithm integrated in One Lambda Fusion software, One Lambda, Inc., Canoga Park, CA, United States) based on donors’ and recipients’ HLA 11-locityping results. Due to intermediate resolution results, most common alleles were used for molecular matching (eplet matching). All eplet mismatches were accepted equally regardless of whether they were verified by antibodies or not. For the calculation of the number of eplets, interlocus class II eplets have been removed. All eplet information was concordant with the Epitope Registry [HLA Epitope Registry (HLA Epitope Registry.com.br, version 3.0)]. Eplets with a high ElliPro score, according to the Epitope registry, were counted as highly immunogenic eplets.

### Clinical Outcomes

The primary clinical parameters for the study were ACR, AMR, CLAD and survival.

ACR was diagnosed by graft biopsy and graded according to the ISHLT classification system [13]. A transplant biopsy was performed routinely after 4 weeks, after 3 months, and after 6 months, as part of the follow up examinations, and on demand in case of clinical suspicion. All grades were treated with steroid pulse therapy starting from A1.

AMR was defined according to the ISHLT consensus report and staged into clinical- and subclinical, and possible and probable AMR [14]. Diagnosis was based on allograft function, conspicuous features in histology such as infiltration with neutrophile granulocytes, positive immunohistochemical C4d staining, development of dnDSA and after exclusion of secondary causes.

CLAD was diagnosed and staged according to the CLAD consensus definition of the ISHLT Guidelines of 2019 [15]. CLAD was characterized by a persistent decline of FEV1 to 80% of baseline or below after exclusion and adequate treatment of secondary causes such as infection, acute cellular or antibody-mediated rejection, or airway stenosis according to current definitions.

### Statistical Analysis

We reported categorical variables as absolute and relative frequencies and numerical variables as means with standard deviation (sd). We compared differences in frequencies and mean values between groups using Chi^2^ or Fisher’s exact test (cell-numbers < 6), and Student’s t-tests, respectively. In the univariate analysis, we used Kaplan-Meier curves with LogRank-test to compare time to ACR, CLAD, and death between patients with and without HLA-DQ-dnDSA. In the multivariate analysis we Cox regression models to analyse time to event data concerning development of ACR, CLAD, and survival. All regression models were adjusted for age, sex, blood type, and CMV risk combination status. Results from regression analysis are reported as Hazard ratios (HR). Statistical significance in all analysis was determined using two-sided *p*-values with alpha errors of <0.05. Data analysis was performed using R Version 4.0.0 and RStudio Version 1.4. Tables and figures were created in RStudio and Microsoft Excel.

## Results

### Study Population

A total of 608 patients underwent lung transplantation between 2012 and 2020. For 220 of these patients, complete HLA 11-locityping of donor and recipient were available. Of these, one patient was excluded due to missing HLA antibody follow up data, 32 patients due to positive HLA antibody status before transplantation, and four patients due to no HLA-class I or HLA-class II mismatches. Finally, we were able to include 183 patients in our study. Recipients and donor characteristics are shown in Table 1. Of the 183 patients included in the study, 62.3% of recipients were male with mean age of 51.8 (sd = 13.0) years. The most common diagnosis was chronic obstructive pulmonary disease (24.6%). Eighty-five percent of patients underwent a double lung transplantation. The majority of lung donors (78.1%) revealed two HLA-DQ mismatches. Only in 1.6% of cases there was no HLA-DQ mismatch between donor and recipient.

**TABLE 1:** Characteristics of study population stratified by HLA-DQ-dnDSA.

	all patients (n = 183)		HLA-DQ-dnDSA (n = 42)		no HLA-DQ-dnDSA (n = 141)		*p*-value
	mean	sd	mean	sd	Mean	sd	
Age in years	51.8	13.0	50.6	12.9	52.1	13.0	0.51
BMI	23.1	4.5	23.7	4.7	22.9	4.4	0.30

	n	%	n	%	n	%	
Sex							
Female	69	37.7%	17	40.5%	52	36.9%	0.81
Male	114	62.3%	25	59.5%	89	63.1%	
Underlying condition							
COPD	45	24.6%	9	21.4%	36	25.5%	0.22
CF	35	19.1%	7	16.7%	28	19.9%	
ILF	26	14.2%	3	7.1%	23	16.3%	
Other (PPH, LAL, EAA, bronchiectasis, sarcoidosis)	77	42.1%	23	54.8%	54	38.3%	
Type of surgery							
Single lung	28	15.3%	4	9.5%	24	17.0%	0.35
Double lung	155	84.7%	38	90.5%	117	83.0%	
Blood type							
O	73	39.9%	21	50.0%	52	36.9%	0.45
A	77	42.1%	15	35.7%	62	44.0%	
B	29	15.8%	5	11.9%	24	17.0%	
AB	4	2.2%	1	2.4%	3	2.1%	
CMV							
R-D-	42	23.0%	7	16.7%	35	24.8%	0.51
R-D+	59	32.2%	15	35.7%	44	31.2%	
R+D-	33	18.0%	10	23.8%	23	16.3%	
R+D+	49	26.8%	10	23.8%	39	27.7%	

Notes: Baseline characteristics of our study collective of lung transplanted patients, stratified by development of *de novo* donor specific antibodies against HLA-DQ during the first year after transplantation. CMV serostatus was determined by ELISA. Categorical variables are reported as absolute and relative frequencies and numerical variables as means with standard deviation. *P*-values between frequencies and mean values between patients with HLA-DQ-dnDSA and patients without are from Chi^2^ and Fisher’s exact test (cell-numbers <6), and Students’ t-test.

BMI, body mass index; CF, cystic fibrosis; CMV, cytomegalovirus; COPD, chronic obstructive pulmonary disease; D, donor; dnDSA, *de-novo* donor-specific antibody; ELISA, enzyme-linked immunosorbent assay; EAA, exogenous allergic alveolitis; HLA, human leukocyte antigen; HLA-DQ-dnDSA, donor specific antibodies against HLA-DQ; ILF, idiopathic lung fibrosis; PPH, primary pulmonary hypertension; sd, standard deviation; R, recipient.

### HLA-Antibody Development

Of the 183 patients, 52 (28.4%) developed dnDSA during 1 year after transplantation. Of all patients with dnDSA, 22/52 (42.3%) patients developed dnDSA against HLA-class I, and 45/52 patients (86.5%) against HLA class II. As previously described by Kleid L. et al [9] we evaluated molecular matching algorithms regarding the development of class I and class II antibodies. However, the main analysis in this paper focuses on HLA-DQ antibodies as the detected class II antibodies were predominantly directed against HLA-DQ (n = 42/45). Among the patients with HLA-DQ-dnDSA, the majority developed antibodies against HLA-DQ3 (59.5%). Most of the patients were immunised against more than one HLA-DQ antigen. The dnDSA characteristics of each patient is listed in the Supplementary Material 1.

### Risk Factors for HLA-DQ-dnDSA Development

There was no significant difference between the number of HLA DQ-antigen mismatches regarding HLA-DQ-dnDSA development. The number of HLA-DQ epMM, as well as the number of highly immunogenic HLA-DQ epMM, was significantly higher in patients who developed HLA-DQ-dnDSA (Table 2).

**TABLE 2:** Comparison between number of antigen- and epletMM.

	All patients (n = 183)		HLA-DQ-dnDSA (n = 42)		no HLA-DQ-dnDSA (n = 141)		*p*-value
	mean	sd	mean	sd	mean	sd	
# HLA-DQ-epMM	14.5	6.8	18.0	6.6	13.5	6.6	0.0001
# highly immunogenic HLA-DQ-epMM	11.1	5.5	14.1	4.7	10.2	5.4	<0.0001

	n	%	n	%	n	%	
HLA-DQ antigenMM							
0	3	1.6%	0	0.0%	3	2.1%	0.71
1	37	20.2%	7	16.7%	30	21.3%	
2	143	78.1%	35	83.3%	108	76.6%	

Notes: Antigen mismatch between donor and recipient was calculated by comparing their HLA-typing results. The amount of epMM was calculated with HLAMatchmaker algorithm (OneLambda Fusion software). Eplets with high ElliPro Scores according to the HLA Epregistry 3.0 were taken into account for the number of highly immunogenic eplet mismatches. The results were stratified by development of HLA-DQ-dnDSA. Mean values were compared using two sided *p*-values from Students’ t-test.

EpMM, eplet mismatches; HLA, human leukocyte antigen; HLA-DQ-dnDSA, donor specific antibodies against HLA-DQ; sd, standard deviation; # = number.

According to our data, recipients who were homozygous for HLA-DQ were significantly more likely to develop HLA-DQ-dnDSA compared to HLA-DQ heterozygous recipients (52.4% vs. 47.6%, *p*-value = 0.03). HLA-DQ1 homozygous recipients transplanted with HLA-DQ3/DQ1 donors were at a higher risk to develop HLA-DQ-dnDSA than patients transplanted with donors of other genotypes (21.4%, *p*-value <0.0001). Absolute and relative frequencies and *p*-values of allele combinations in homozygous recipients stratified by HLA-DQ-dnDSA are summarized in Table 3. If both, recipient and donor, had the allele combination DQ3/DQ1 this was significantly associated with not having HLA-DQ-dnDSA (0.0% vs. 12.1%, *p*-value = 0.01).

**TABLE 3:** Association between recipient/donor HLA-DQ alleles and development of HLA-DQ-dnDSA.

	HLA-DQ-dnDSA (n = 42)		no HLA-DQ-dnDSA (n = 141)		*p*-value
	n	%	n	%	
Recipient alleles					
homozygous HLA-DQ	22	52.4	45	31.9	
heterozygous HLA-DQ	20	47.6	96	68.1	0.03
Allele combination of HLA-DQ-homozygous patients					
**rec:** DQ1, DQ1 **do:** DQ1, DQ1	1	2.4	10	7.1	0.46
**rec:** DQ1, DQ1 **do**: DQ2, DQ1	0	0.0	3	2.1	1.00
**rec:** DQ1, DQ1 **do:** DQ2, DQ2	0	0.0	1	0.7	1.00
**rec:** DQ1, DQ1 **do:** DQ2, DQ3	1	2.4	2	1.4	0.54
**rec:** DQ1, DQ1 **do:** DQ3, DQ1	9	21.4	2	1.4	<0.0001
**rec:** DQ1, DQ1 **do:** DQ3, DQ3	2	4.8	4	2.8	0.62
**rec:** DQ1, DQ1 **do:** DQ4, DQ1	0	0.0	1	0.7	1.00
**rec:** DQ2, DQ2 **do**: DQ1, DQ1	-0	0.0	1	0.7	1.00
**rec:** DQ2, DQ2 **do**: DQ2, DQ3	1	2.4	1	0.7	0.41
**rec:** DQ2, DQ2 **do:** DQ3, DQ1	0	0.0	3	2.1	1.00
**rec:** DQ3, DQ3 **do:** DQ1, DQ1	1	2.4	5	3.5	1.00
**rec:** DQ3, DQ3 **do:** DQ2, DQ1	2	4.8	1	0.7	0.13
**rec**: DQ3, DQ3 **do:** DQ2, DQ2	0	0.0	1	0.7	1.00
**rec:** DQ3, DQ3 **do:** DQ2, DQ3	2	4.8	1	0.7	0.13
**rec**: DQ3, DQ3 **do**: DQ3, DQ1	2	4.8	7	5.0	1.00
**rec:** DQ3, DQ3 **do:** DQ3, DQ3	1	2.4	1	0.7	0.41
**rec:** DQ3, DQ3 **do:** DQ3, DQ4	0	0.0	1	0.7	1.00

Notes: The cohort was analysed according to patients and donors HLA-DQ typing. Certain cross-reactive allele groups were combined into one group as follows: HLA-DQ1 = HLA-DQ5/DQ6; HLA-DQ3 = HLA-DQ7/DQ8/DQ9. The results were stratified by development of HLA-DQ-dnDSA. *P*-values were derived from Chi^2^ test or Fisher’s exact test (cell numbers < 6).

rec, recipient; do, donor; HLA-DQ-dnDSA, donor specific antibodies against HLA-DQ.

The following HLA-DQ eplets were significantly more prevalent in the HLA-DQ-dnDSA group: 55PP (50.0% vs. 22.7%, *p*-value = 0.001), 55PPD (47.6% vs. 23.4%, *p*-value = 0.004), 66ER (47.6% vs. 22.7%, *p*-value = 0.003), 182N (45.2% vs. 22.7%, *p*-value = 0.01), 70RT (45.2% vs. 21.3%, *p*-value = 0.004), 45EV (47.6% vs. 19.9%, *p*-value = 0.001), 167H (45.2% vs. 20.6%, *p*-value = 0.003), 66IL (26.2% vs. 12.1%, *p*-value = 0.05), 61FT (28.6% vs. 9.9%, *p*-value = 0.005), 84QL (28.6% vs. 9.2%, *p*-value = 0.003). The absolute and relative frequencies and *p*-values for these “high-risk eplets” are shown in Table 4. The eplet 130Q was significantly more prevalent in patients without HLA-DQ-dnDSA (Supplementary Material 2).

**TABLE 4:** Description of “high-risk” eplets.

eplet	Polymorphic AA residues	Main alleles (most common)	ElliPro Score	HLA-DQ-dnDSA (n = 42)		no HLA-DQ-dnDSA (n = 141)		*p*-value
				n	%	n	%	
55PP	55P56P	DQ3 (DQB1*03:01, DQB1*03:02, DQB1*03:03)	High	21	50.0	32	22.7	0.001
55PPD	55P56P57P	DQ3 (DQB1*03:01, DQB1*03:03)	High	20	47.6	33	23.4	0.004
66ER	66E67V70R71T	DQ3 (DQB1*03:01, DQB1*03:02, DQB1*03:03) scattered on DQ1 (DQB1*06:04 DQB1*06:05, DQB1*06:06)	High	20	47.6	32	22.7	0.003
182N	182N	DQ3 (DQB1*03:01, DQB1*03:02, DQB1*03:03), DQ4	High	19	45.2	32	22.7	0.01
70RT	70R71T	DQ3 (DQB1*03:01, DQB1*03:02, DQB1*03:03) scattered on DQ1 (DQB1*06:01 DQB1*06:04 DQB1*06:05, DQB1*06:06)	High	19	45.2	30	21.3	0.004
45EV	45E46V47Y	DQ3 (DQB1*03:01)	High	20	47.6	28	19.9	0.001
167H	167H	DQ3 (DQB1*03:01), DQ1 (DQB1*06:01)	High	19	45.2	29	20.6	0.003
66IL	66I69L	DQA1*02,03,05	Intermediate	11	26.2	17	12.1	0.05
61FT	61F64T55R	several DQA-alleles (except DQA1*01)	High	12	28.6	14	9.9	0.005
84QL	84Q86E87L89T90T125A	DQ3 (DQB1*03:01, DQB1*03:02, DQB1*03:03), DQ2, DQ4	High	12	28.6	13	9.2	0.003

Notes: List of eplets that were significantly associated with the development of *de novo* HLA-DQ-dnDSA, including their properties such as polymorphic amino acid residues, representing alleles and ElliPro scores according to the HLA Epitope Registry (HLA Epitope Registry.com.br). *P*-values were derived from Chi^2^ test or Fisher’s exact test (cell numbers < 6).

AA, amino acid; HLA-DQ-dnDSA, *de-novo* donor-specific antibodies against HLA-DQ; … = also represented on other rare alleles.

### HLA-DQ-dnDSA and Clinical Outcomes

Among the 183 patients included in our study, 58 patients suffered from ACR. Of these, 18 patients were positive for HLA-DQ-dnDSA. A total of 10 patients with diagnosed ACR died, three of them within the first year after transplantation.

Within our cohort, 52 patients showed signs of a possible or probable AMR. Of these, 47 were staged as subclinical and five patients as possible clinical AMR. Among the five patients with clinical AMR, there were 3 patients with severe outcome who died within the first year; all had HLA-DQ-dnDSA. Patients with dnDSA (class I, class II, HLA-DQ) showed significantly more clinical and subclinical AMR (Table 5).

**TABLE 5:** Association of dnDSA with antibody-mediated rejection.

	Class-I-DSA (n = 22)		No class-I-DSA (n = 161)		*p*-value
	%		n	%	
AMR					
Yes	18	81.8%	34	21.1%	<0.0001
No	4	18.2%	127	78.9%	
AMR subtype					
None	4	18.2%	127	78.9%	<0.0001
1a	3	13.6%	2	1.2%	
2a	15	68.2%	30	18.6%	
2b	0	0.0%	2	1.2%	

	Class-II-DSA (n = 45)		No class-II-DSA (n = 138)		*p*-value
	%		n	%	
AMR					
Yes	32	71.1%	20	14.5%	<0.0001
No	13	28.9%	118	85.5%	
AMR subtype					
None	13	28.9%	118	85.5%	<0.0001
1a	5	11.1%	0	0.0%	
2a	25	55.6%	20	14.5%	
2b	2	4.4%	0	0.0%	

	DQ-DSA (n = 42)		No DQ-DSA (n = 141)		*p*-value
	%		n	%	
AMR					
Yes	29	69.0%	23	16.3%	<0.0001
No	13	31.0%	118	83.7%	
AMR subtype					
None	13	31.0%	118	83.7%	<0.0001
1a	4	9.5%	1	0.7%	
2a	23	54.8%	22	15.6%	
2b	2	4.8%	0	0.0%	

Notes: Overview of patients with diagnosed with antibody-mediated rejection classified according to the ISHLT consensus guidelines and stratified by development of HLA class I, class II or HLA-DQ-dnDSA.

AMR, antibody-mediated rejection; ISHLT, the international society of heart and lung transplantation; 1a = possible clinical antibody-mediated rejection; 2a = possible subclinical antibody-mediated rejection, 2b = probable subclinical antibody-mediated rejection; HLA, human leucocyte antigen; HLA-DQ-dnDSA, *de-novo* donor-specific antibodies against HLA-DQ.

Concerning long term outcome, 35 patients were diagnosed with CLAD of which 12 were positive for HLA-DQ-dnDSA. A total of 7 patients died, all of them after more than 1 year after transplantation.

### Kaplan-Meier Curves

**FIGURE 1:**
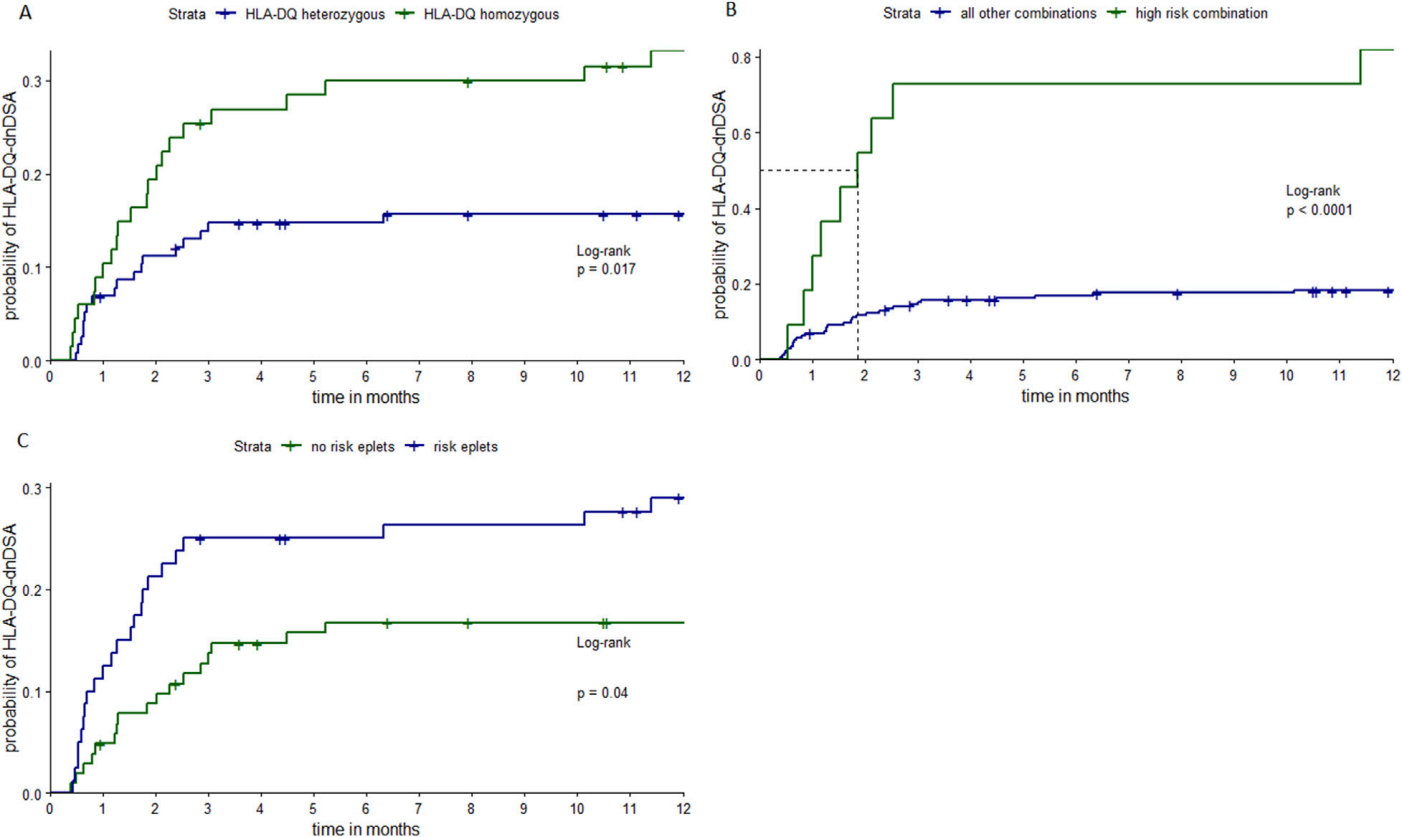
Kaplan-Meier curves stratified by different risk-factors. Notes: Kaplan-Meier curves of time until first detection of HLA-DQ-dnDSA, stratified by being homozygous or heterozygous for HLA-DQ **(A)**, by having the high-risk allele combination (homozygous HLA-DQ1 patients, recipients transplanted with HLA-DQ3/DQ1 typed donors) or any other allele combination **(B)** and stratified by the presence of one or more high-risk eplets **(C)**. Patients and donors who were homozygous either for HLA-DQ5 and/or -DQ6 were combined as HLA-DQ1 homozygous, patients homozygous for either HLA-DQ7, -DQ8 and/or DQ9 were termed as HLA-DQ3 homozygous. The following eplets are determined as high-risk eplets 55PP, 55PPD, 66ER, 182N, 70RT, 45EV, 167H, 66IL, 61FT, 84QL. *P*-values from LogRank test. HLA, human leucocyte antigen; HLA-DQ-dnDSA, *de-novo* donor-specific antibodies against HLA-DQ locus.

Figure 1 shows Kaplan-Meier curves of time until first detection of HLA-DQ-dnDSA, by being homozygous for HLA-DQ, by having a high-risk allele combination (homozygous for HLA-DQ1 in combination with HLA-DQ3/DQ1 donors), and by having at least one high-risk eplet mismatch. Patients with these risk-factors had a significantly higher risk to develop HLA-DQ-dnDSA compared to patients without.

**FIGURE 2:**
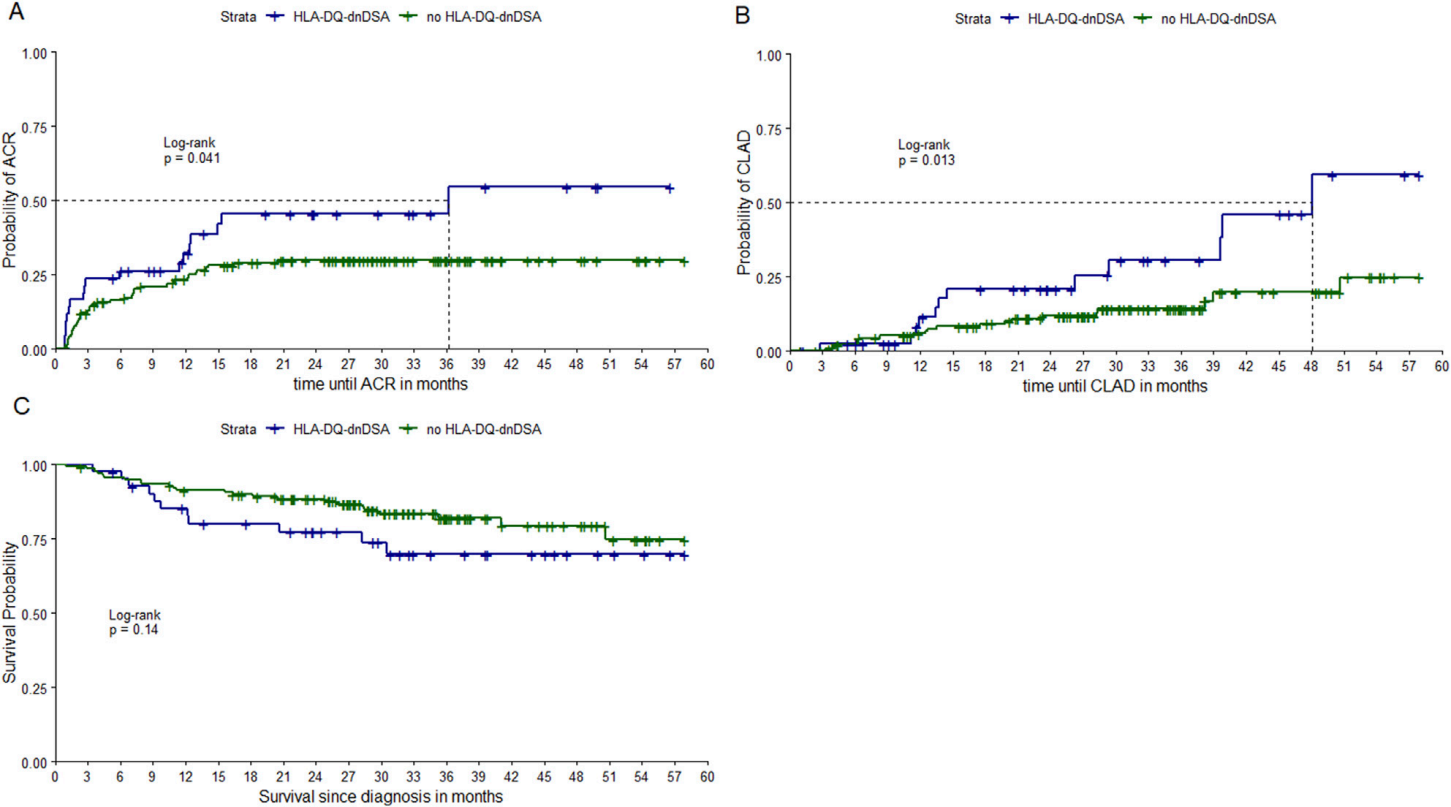
Kaplan Meier curves stratified by HLA-DQ-dnDSA. Notes: Kaplan-Meier curves of time until ACR, time until CLAD and time until death, stratified by development of HLA-DQ-dnDSA. *P*-values from LogRank test. ACR, acute cellular rejection; CLAD, chronic allograft dysfunction; HLA-DQ-dnDSA, *de-novo* donor-specific antibodies against HLA-DQ locus. Notes: Kaplan-Meier curves of time until ACR **(A)**, time until CLAD **(B)** and time until death **(C)**, stratified by development of HLA-DQ-dnDSA.

Figure 2 shows Kaplan-Meier curves of time until ACR, CLAD, and death stratified by development of HLA-DQ-dnDSA. We found that having HLA-DQ-dnDSA was significantly associated with time to ACR (*p*-value = 0.04) and time to CLAD (*p*-value = 0.01). However, we did not find a significant association between HLA-DQ-dnDSA and overall survival (*p*-value = 0.14).

Even though our study focused on HLA-DQ-dnDSA we additionally analysed HLA class I and class II antibodies with clinical outcome data. The results of this analysis displayed as Kaplan-Meier curves can be found in the Supplementary Material 3, 4. HLA class I dnDSA were not significantly associated with clinical outcomes, HLA class II dnDSA were significantly associated with time to CLAD.

### Multivariate Regression Analysis

The multivariate Cox regression models of time to HLA-DQ-dnDSA confirmed the significant association between homozygosity of HLA-DQ (model 1), and the high-risk allele combination (model 2) and the development of HLA-DQ-dnDSA from the univariate analysis. In contrast to the presence of at least one high-risk eplet (model 3) the number of high-risk eplets was still significantly associated with time to HLA-DQ-dnDSA (model 4).

In the multivariate Cox regression, no direct correlation was found between the above-mentioned risk factors and survival or time to ACR. However, the high-risk allele combination was significantly associated with time to CLAD.

The association of HLA-DQ-dnDSA with time to ACR (HR = 1.85, *p*-value = 0.04) and time to CLAD (HR = 2.61, *p*-value = 0.01) revealed significant results. Survival time and HLA-DQ-dnDSA were not significantly associated. Table 6 shows results of all regression models.

**TABLE 6:** Results of regression analyses.

		Beta	HR	se	z-value	*p*-value
Cox regression of time to HLA-DQ-dnDSA						
Model 1	Recipient allele homozygous vs. Heterozygous	0.68	1.97	0.32	2.10	0.04
Model 2	High risk allele combination	1.82	6.17	0.39	4.61	<0.0001
Model 3	High risk eplet yes vs. No	0.57	1.76	0.32	1.78	0.08
Model 4	# of high risk eplets	0.16	1.17	0.04	3.97	<0.0001
Cox regression of time to CLAD						
Model 5	Recipient allele homozygous vs. Heterozygous	−0.58	0.56	0.42	−1.40	0.16
Model 6	High risk allele combination	1.15	3.15	0.56	2.04	0.04
Model 7	High risk eplet yes vs. No	0.14	1.15	0.36	0.39	0.70
Model 8	# Of high risk eplets	0.04	1.04	0,05	0.92	0.36
cox regression of ACR, CLAD, and survival						
Model 9	HLA-DQ-dnDSA and ACR	0.62	1.85	0.29	2.14	0.03
Model 10	HLA-DQ-dnDSA and CLAD	0.96	2.61	0.38	2.55	0.01
Model 11	HLA-DQ-dnDSA and survival	0.61	1.83	0.38	1.62	0.11

Notes: Results from Cox regression analysis of development of HLA-DQ-dnDSA (models 1–4), time to CLAD recipient allele homozygous/heterozygous (model 5), high-risk allele combination (model 6), high-risk eplet yes vs. no (model 7), #of high risk eplets (model 8), and Cox regression analysis of time to ACR (model 9), CLAD (model 10) and survival (model 11). All Cox regression models are adjusted by age, sex, and CMV risk combination.

ACR, acute cellular rejection; CLAD, chronic lung allograft dysfunction; CMV, cytomegalovirus; HLA, human leucocyte antigen; HLA-DQ-dnDSA, de-novo donor-specific antibodies against HLA-DQ; OR, odds ratio; se = standard error; # = number.

## Discussion

In this study, we analysed 183 lung transplant patients for their HLA-antibody status and the impact of the presence of HLA-DQ-dnDSA on their clinical outcomes. We used antigen- and eplet-based HLA-matching as a new approach in addition to determining quantitative amount of mismatches for risk assessment. Our purpose is to add valuable information to the growing knowledge about risk factors for dnDSA and rejection after lung transplantation. In addition, our results indicate an easy applicable approach to identify high risk patient donor combinations that can be used in clinical practice.

We found that patients with HLA-DQ-dnDSA were at a significantly higher risk for developing ACR and CLAD. This finding further supports the association of HLA-DQ-dnDSA, with ACR, CLAD, and overall survival found in other studies [16–18]. Our results also strengthen the association of class I, class II and HLA-DQ-dnDSA with AMR, which is confirmatory in nature as dnDSA are in most cases one of the diagnosis criteria for AMR. We could not confirm the effects on survival, which might be due to a shorter follow-up period. However, our results are in line with Ennis et al, who investigated the impact of *de novo* HLA-DQ antibodies resulting from a DQA1*05 + DQB1*02/DQB*03:01 mismatch, and showed that these dnDSA are associated with CLAD but not survival [19].

A few studies indicate that HLA-DQ-dnDSA are the most prevalent in cardiothoracic transplant patients and there is evidence of inferior graft outcomes [16, 20]. This has also been described in renal [21] and cardiac transplantation [22]. Increased expression of HLA-class II molecules in inflamed lung tissue might be one explanation [23, 24]. The development of an ACR can affect long-term complications such as the development of CLAD and have a negative impact on patients’ survival. Lowering the immunological risk for developing HLA-DQ-dnDSA and therefore the risk for ACR, AMR and CLAD will contribute to improve graft outcome.

Regarding antigen-based HLA-matching, we could show that the number of antigen mismatches does not play a major role. Rather, it is important to look a bit closer at the patient’s own HLA-DQB1 typing. We were able to demonstrate that HLA-DQ-homozygous patients have a significantly higher risk to develop HLA-DQ-dnDSA than HLA-DQ heterozygous patients. The risk of developing HLA-DQ-dnDSA was 2.34 times higher compared to heterozygous patients. HLA-DQ homozygosity is a risk factor as these patients are facing more structural differences than heterozygous patients. One specific recipient-donor antigen combination, recipient homozygous for HLA-DQ1 with HLA-DQ1/DQ3 donors, was significantly associated with the development of HLA-DQ-dnDSA. This high-risk donor recipient constellation was also described in the publication of McCaughan et al [20]. They showed similar findings within their patient cohort and assume electrostatic potentials as a possible explanation for the increased immunogenicity. They also described that a combination of the foreign HLA-DQA1 and HLA-DQB1 structures could be crucial for immunisation. Besides confirming the aforementioned risk-constellation, the added value of our study is reflected in the clinical outcome parameters of our patient cohort. Unfortunately, we were not able to compare the allele combinations on high resolution as the number of combinations was too high and the number of patients within each combination too small. Although one of the strengths of our study is the large patient cohort, the number of patients with HLA-DQ-dnDSA was small, especially for the analysis of high-risk donor-recipient combinations. Further research on this topic and larger cohorts might help to see whether more high risk or low risk combinations can be revealed.

Regarding HLA eplet matching, we were able to show that the number of epMM was associated with the development of HLA-DQ-dnDSA. Previously we had shown that it was associated with the development of HLA-antibodies [11]. Similar results have also been reported by Hiho et al. [25]. Both works show that comparing the number of molecular mismatches can be an approach for risk stratification in lung transplantation. One limitation of using and comparing eplet matching data are uncertainties not only in terminology but also in their application, as recently described and summarized by Tambur et al [26]. Depending on user preferences and different versions of the HLAMatchmaker algorithm, eplet matching results or eplets loads can lead to discordant results concerning number and type of mismatched eplets. In our study, eplets designated as “antibody-confirmed” and those lacking confirmation were treated equally due to the fluid nature of classification. An eplet labelled as unverified presently could potentially undergo experimental validation by a research team in the foreseeable future. Moreover, the validation process lacks clear regulation and consistency, making comparisons challenging [27]. Tambur et al. also point out that the number of eplet mismatches should be considered with caution. Combining recipient and donor eplets into one so called HLAMatchmaker “eplet universe” and not considering individual alleles, bears the risk of creating potential ambiguities and the immunologic validity of this concept still needs to be determined. Therefore, it is important to also look at specific eplets. In this study we identified potential high-risk eplets that have a greater potential to induce the development of dnDSA. Our findings are similar to the data of Schawalder et al, who analysed child-specific anti-HLA DQ-antibodies after pregnancy [28]. They found that the eplets 55PP and 45EV are highly reacting eplets and we were able to confirm these findings within our lung transplant cohort, and additionally identified several more high-risk eplets. Hereby, one must bear in mind that different versions of the Matchmaker have been used (2.1 and 3.0). There were also patients with high-risk epMMs who did not develop HLA-DQ-dnDSA in our cohort. When we compared these patients to patients with high-risk epMM and HLA-DQ-dnDSA, we found that being female slightly elevated the risk for immunisation. This might be explained by prior contact to foreign HLA during pregnancies. Immunisation is a multifactorial process and adherence to immunosuppressive therapy might also be a factor. It would be interesting to monitor patients’ compliance in further studies. Nevertheless, we clearly identified several high-risk eplets that were significantly more immunogenic than others which in most cases resulted from a HLA-DQB1*03:01 mismatch. Snanoud and colleagues reported similar findings in their kidney transplant cohort [29].

As described by Schawalder et al., one major limitation in defining immunogenicity of eplets is to identify the true target of the antibody. Each HLA-mismatch leads to a set of overlapping eplets, each eplet on its own or several eplets might explain the reaction pattern in the Luminex Assay [28]. Especially the HLA-DQ locus is very complex as it is composed of an α and β chain, each carrying individual immunogenic eplets. Moreover, a distinction between anti-HLA-DQA1 and -DQB1 antibodies in Luminex data interpretation is sometimes not possible. Most of our high-risk eplets were derived from donor´s HLA-DQB1, however in their cohort of lung transplant patients González-López et al showed that also HLA-DQA1 epMM could lead to inferior graft outcomes [30].

One limitation of our study is the resolution of our HLA typings. Analysis with high-resolution typing and comparing donor and recipient on the amino acid level might help to reveal the true antibody targets. Available typing information has improved over the last years and hopefully studies with more recent high-resolution typing data will help to better perform eplet analysis and make molecular mismatch methods more accurate.

Although further research on this topic is necessary, there is a clear tendency towards HLA-DQB1*03:01 as a highly immunogenic HLA mismatch, regarding both the antigen and the responsible eplets. Randomised clinical trials are needed to gain a better understanding of the clinical relevance and potentially the significance of increased immunosuppression in a high-risk constellation.

## Conclusion

Specific HLA-DQ mismatches seem to be particularly responsible for the development of *de novo* HLA-antibodies after lung transplantation, which in return result in inferior graft outcomes. EpMM analysis might be a helpful tool for risk assessment in order to support clinicians in identifying patients at higher risk for HLA-DQ-dnDSA. Although it will take some time until molecular matching algorithms will be ready to be consistently used in clinical routine, our analysis has highlighted HLA-DQ phenotypes of high-risk recipients, recipient-donor combinations and high-risk eplets for risk-assessment. With this early information about increased humoral risk, adjustment of immunosuppression or closer follow-up could lead to improved long-term survival in lung transplant patients.

## Data Availability

The raw data supporting the conclusions of this article will be made available by the authors, without undue reservation.
