# Supplementary material for: High-Risk HLA-DQ Mismatches Are Associated With Adverse Outcomes After Lung Transplantation
Source: Transpl Int. 2024 Sep 19;37:13010. doi: 10.3389/ti.2024.13010 (PMC11460317; doi:10.3389/ti.2024.13010)

**Supp.Mat. 1 - Patients immunization characteristics**

| Patient | dnDSA class | dnDSA cumulative | persistent/transient | peak MFI |
| --- | --- | --- | --- | --- |
| 001 | class II | DQ7 | persistent | 3568 |
| 002 | class II | DP13 | transient | 3888 |
| 003 | class I + II | Cw7 DQ6 | persistent | 4350 |
| 004 | class II | DQ7 | transient | 2361 |
| 005 | class II | DQ7 | persistent | 11082 |
| 006 | class II | DQ7 | transient | 5651 |
| 007 | class II | DQ9 | persistent | 10131 |
| 008 | class I | A1 | transient | 2115 |
| 009 | class II | DQ6 | persistent | 3583 |
| 010 | class II | DQA1*05:01 | transient | 2389 |
| 011 | class I + II | A1 B51 Cw7 DR7 DQ2 | persistent | 10849 |
| 012 | class II | DQA1*05:01 | persistent | 4059 |
| 013 | class I + II | B44 DP9 | persistent | 3188 |
| 014 | class II | DQ5 | persistent | 6426 |
| 015 | class II | DQA1*05:01 | persistent | 3103 |
| 016 | class II | DR51 DQ5 | persistent | 14232 |
| 017 | class I + II | Cw5 DR4 DQ2 DQ8 | persistent | 17724 |
| 018 | class II | DR7 DQ2 | persistent | 3833 |
| 019 | class II | DQ7 | persistent | 23341 |
| 020 | class II | DQ7 | persistent | 5704 |
| 021 | class I + II | B7 DQ7 | persistent | 11104 |
| 022 | class II | DR53 DQ7 | transient | 23423 |
| 023 | class II | DQ2 DQ6 | persistent | 9022 |
| 024 | class II | DQ7 | persistent | 4752 |
| 025 | class I | A2 | persistent | 1134 |
| 026 | class I | Cw7 | persistent | 1410 |
| 027 | class I | A24 | persistent | 5081 |
| 028 | class II | DQ5 | persistent | 2538 |
| 029 | class II | DQ6 DQA1*05:01 | persistent | 19805 |
| 030 | class II | DQ5 DQ6 | persistent | 12427 |
| 031 | class I + II | B8 DQA1*05:01 | persistent | 11893 |
| 032 | class II | DQ7 | persistent | 9515 |
| 033 | class II | DQ2 | persistent | 5796 |
| 034 | class I + II | B57 DR4 DQ7 | transient | 8312 |
| 035 | class II | DQ7 | persistent | 7034 |
| 036 | class I | Cw4 | persistent | 5068 |
| 037 | class II | DQ8 | persistent | 5930 |
| 038 | class I + II | A2 DR53 | persistent | 3376 |
| 039 | class I | B58 | persistent | 1359 |
| 040 | class I | A24 | persistent | 3152 |
| 041 | class II | DQ7 | persistent | 6221 |
| 042 | class II | DQ7 | persistent | 5215 |
| 043 | class II | DQ7 | persistent | 13183 |
| 044 | class I + II | A2 Cw6 DR7 DQ9 | persistent | 11489 |
| 045 | class II | DQ7 | transient | 5738 |
| 046 | class II | DQ6 DQ7 | persistent | 2852 |
| 047 | class I + II | A2 DQ2 | transient | 4163 |
| 048 | class I + II | B60 DQ8 | persistent | 7343 |
| 049 | class I + II | A1 A23 B8 DQ8 | persistent | 6273 |
| 050 | class I + II | A2 DQ7 DQ8 | persistent | 6817 |
| 051 | class I + II | B8 DQA1*05:01 | persistent | 6898 |
| 052 | class I + II | Cw3 DQ2 DQ7 | persistent | 14995 |

Notes: Immunization characteristics of all patients with dnDSA with additional information about peak MFI value of Single-Antigen Bead results. MFI values reported have been normalized with negative bead values. Transient antibodies have been measured once, whereas persistent antibodies have been detected more than once.

dnDSA = de-novo donorspecific HLA-antibody, MFI = mean fluorescence intensity

**Supp. Mat. 2 - Eplet frequencies**

|  | all patients  (n = 183) | | HLA-DQ-dnDSA  (n = 42) | | no HLA-DQ-dnDSA  (n = 141) | | p-value |  |
| --- | --- | --- | --- | --- | --- | --- | --- | --- |
| eplet | n | % | n | % | n | % |  |  |
| 77R | 56 | 30.6% | 18 | 42.9% | 38 | 27.0% | 0.08 |  |
| 175E | 54 | 29.5% | 15 | 35.7% | 39 | 27.7% | 0.42 |  |
| 55PP | 53 | 29.0% | 21 | 50.0% | 32 | 22.7% | 0.001 |  |
| 55PPD | 53 | 29.0% | 20 | 47.6% | 33 | 23.4% | 0.004 |  |
| 75V | 53 | 29.0% | 16 | 38.1% | 37 | 26.2% | 0.20 |  |
| 40ERV | 53 | 29.0% | 14 | 33.3% | 39 | 27.7% | 0.60 |  |
| 66ER | 52 | 28.4% | 20 | 47.6% | 32 | 22.7% | 0.003 |  |
| 182N | 51 | 27.9% | 19 | 45.2% | 32 | 22.7% | 0.01 |  |
| 70RT | 49 | 26.8% | 19 | 45.2% | 30 | 21.3% | 0.004 |  |
| 45EV | 48 | 26.2% | 20 | 47.6% | 28 | 19.9% | 0.001 |  |
| 167H | 48 | 26.2% | 19 | 45.2% | 29 | 20.6% | 0.003 |  |
| 25FT | 48 | 26.2% | 14 | 33.3% | 34 | 24.1% | 0.32 |  |
| q30H | 47 | 25.7% | 8 | 19.0% | 39 | 27.7% | 0.36 |  |
| 67VG | 47 | 25.7% | 11 | 26.2% | 36 | 25.5% | 1.00 |  |
| 74S | 46 | 25.1% | 10 | 23.8% | 36 | 25.5% | 0.98 |  |
| 125G | 46 | 25.1% | 9 | 21.4% | 37 | 26.2% | 0.67 |  |
| 86A | 44 | 24.0% | 10 | 23.8% | 34 | 24.1% | 1.00 |  |
| 40GR | 44 | 24.0% | 15 | 35.7% | 29 | 20.6% | 0.07 |  |
| 55RPD | 43 | 23.5% | 10 | 23.8% | 33 | 23.4% | 1.00 |  |
| 70GT | 43 | 23.5% | 11 | 26.2% | 32 | 22.7% | 0.79 |  |
| 87F | 43 | 23.5% | 11 | 26.2% | 32 | 22.7% | 0.79 |  |
| 87Y | 43 | 23.5% | 8 | 19.0% | 35 | 24.8% | 0.57 |  |
| 75S | 43 | 23.5% | 14 | 33.3% | 29 | 20.6% | 0.13 |  |
| q37YV | 42 | 23.0% | 10 | 23.8% | 32 | 22.7% | 1.00 |  |
| 56PA | 42 | 23.0% | 13 | 31.0% | 29 | 20.6% | 0.23 |  |
| 116I | 42 | 23.0% | 10 | 23.8% | 32 | 22.7% | 1.00 |  |
| 185I | 42 | 23.0% | 8 | 19.0% | 34 | 24.1% | 0.63 |  |
| 129QS | 41 | 22.4% | 8 | 19.0% | 33 | 23.4% | 0.70 |  |
| 52PQ | 40 | 21.9% | 9 | 21.4% | 31 | 22.0% | 1.00 |  |
| 66DR | 40 | 21.9% | 13 | 31.0% | 27 | 19.1% | 0.16 |  |
| 75IL | 40 | 21.9% | 13 | 31.0% | 27 | 19.1% | 0.16 |  |
| 52LL | 39 | 21.3% | 13 | 31.0% | 26 | 18.4% | 0.13 |  |
| 56PD | 39 | 21.3% | 8 | 19.0% | 31 | 22.0% | 0.85 |  |
| 52SK | 39 | 21.3% | 9 | 21.4% | 30 | 21.3% | 1.00 |  |
| 66D | 38 | 20.8% | 11 | 26.2% | 27 | 19.1% | 0.44 |  |
| 9F | 37 | 20.2% | 7 | 16.7% | 30 | 21.3% | 0.66 |  |
| 55R | 37 | 20.2% | 8 | 19.0% | 29 | 20.6% | 1.00 |  |
| 47KHL | 37 | 20.2% | 11 | 26.2% | 26 | 18.4% | 0.38 |  |
| 76L | 37 | 20,2% | 11 | 26.2% | 26 | 18.4% | 0.38 |  |
| 67VT | 34 | 18.6% | 9 | 21.4% | 25 | 17.7% | 0.75 |  |
| 125SQ | 34 | 18.6% | 7 | 16.7% | 27 | 19.1% | 0.89 |  |
| q57V | 33 | 18.0% | 5 | 11.9% | 28 | 19.9% | 0.34 |  |
| q76V | 33 | 18.0% | 8 | 19.0% | 25 | 17.7% | 1.00 |  |
| 37YA | 31 | 16.9% | 8 | 19.0% | 23 | 16.3% | 0.86 |  |
| 74EL | 31 | 16.9% | 8 | 19.0% | 23 | 16.3% | 0.86 |  |
| 45GV | 29 | 15.8% | 7 | 16.7% | 22 | 15.6% | 1.00 |  |
| 55PPA | 29 | 15.8% | 7 | 16.7% | 22 | 15.6% | 1.00 |  |
| q26L | 28 | 15.3% | 9 | 21.4% | 19 | 13.5% | 0.31 |  |
| q77T | 28 | 15.3% | 7 | 16.7% | 21 | 14.9% | 0.97 |  |
| 66IL | 28 | 15.3% | 11 | 26.2% | 17 | 12.1% | 0.05 |  |
| 160AD | 27 | 14.8% | 9 | 21.4% | 18 | 12.8% | 0.25 |  |
| 13GM | 26 | 14.2% | 8 | 19.0% | 18 | 12.8% | 0.44 |  |
| 40E | 26 | 14.2% | 6 | 14.3% | 20 | 14.2% | 1.00 |  |
| 61FT | 26 | 14.2% | 12 | 28.6% | 14 | 9.9% | 0.005 |  |
| 84QL | 25 | 13.7% | 12 | 28.6% | 13 | 9.2% | 0.003 |  |
| 182S | 25 | 13.7% | 8 | 19.0% | 17 | 12.1% | 0.37 |  |
| 130A | 23 | 12.6% | 6 | 14.3% | 17 | 12.1% | 0.91 |  |
| 135G | 22 | 12.0% | 7 | 16.7% | 15 | 10.6% | 0.29 |  |
| 75I | 22 | 12.0% | 5 | 11.9% | 17 | 12.1% | 1.00 |  |
| 56PS | 18 | 9.8% | 4 | 9.5% | 14 | 9.9% | 1.00 |  |
| 25YT | 16 | 8.7% | 4 | 9.5% | 12 | 8.5% | 0.76 |  |
| 129H | 16 | 8.7% | 7 | 16.7% | 9 | 6.4% | 0.06 |  |
| 130Q | 15 | 8.2% | 0 | 0.0% | 15 | 10.6% | 0.02 |  |
| 167R | 15 | 8.2% | 5 | 11.9% | 10 | 7.1% | 0.34 |  |
| 160D | 15 | 8.2% | 4 | 9.5% | 11 | 7.8% | 0.75 |  |
| 45G | 14 | 7.7% | 5 | 11.9% | 9 | 6.4% | 0.32 |  |
| 66EV | 11 | 6.0% | 3 | 7.1% | 8 | 5.7% | 0.72 |  |
| q9Y | 7 | 3.8% | 3 | 7.1% | 4 | 2.8% | 0.20 |  |
| 56L | 7 | 3.8% | 0 | 0.0% | 7 | 5.0% | 0.35 |  |
| 66IT | 7 | 3.8% | 2 | 4.8% | 5 | 3.5% | 0.66 |  |
| 46VY | 6 | 3.3% | 1 | 2.4% | 5 | 3.5% | 1.00 |  |
| 3P | 2 | 1.1% | 0 | 0.0% | 2 | 1.4% | 1.00 |  |
| 185T | 2 | 1.1% | 0 | 0.0% | 2 | 1.4% | 1.00 |  |
| 2G | 2 | 1.1% | 1 | 2.4% | 1 | 0.7% | 0.41 |  |
| 3S | 1 | 0.5% | 1 | 2.4% | 0 | 0.0% | 0.23 |  |
| 130R | 1 | 0.5% | 0 | 0.0% | 1 | 0.7% | 1.00 |  |
| 135D | 1 | 0.5% | 0 | 0.0% | 1 | 0.7% | 1.00 |  |
| 160S | 1 | 0.5% | 1 | 2.4% | 0 | 0.0% | 0.23 |  |
|  |  |  |  |  |  |  |  |  |

Notes: List of all eplets stratified by the development of de novo HLA-DQ-dnDSA. P-values were derived from Chi2 test or Fisher’s exact test (cell numbers < 6).

HLA-DQ-dnDSA = de-novo donor-specific antibodies against HLA-DQ locus

**Supp. Mat. 3 – Kaplan Meier curves stratified by class I HLA-dnDSA**


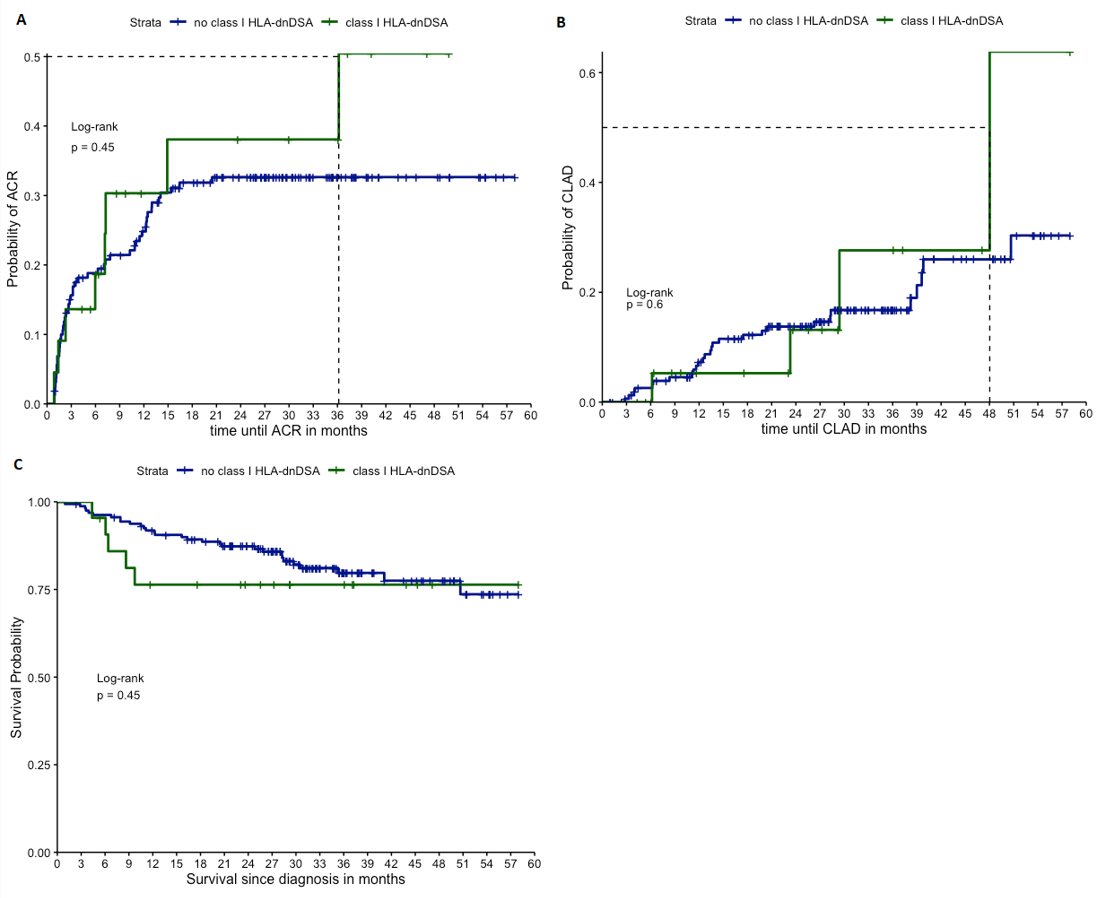


Notes: Kaplan-Meier curves of time until ACR, time until CLAD and time until death, stratified by development of class I HLA-dnDSA. P-values from LogRank test.

ACR = acute cellular rejection, CLAD = chronic allograft dysfunction, HLA-DQ-dnDSA = de-novo donor-specific antibodies against HLA-DQ locus

**Supp. Mat. 4 – Kaplan Meier curves stratified by class II HLA-dnDSA**


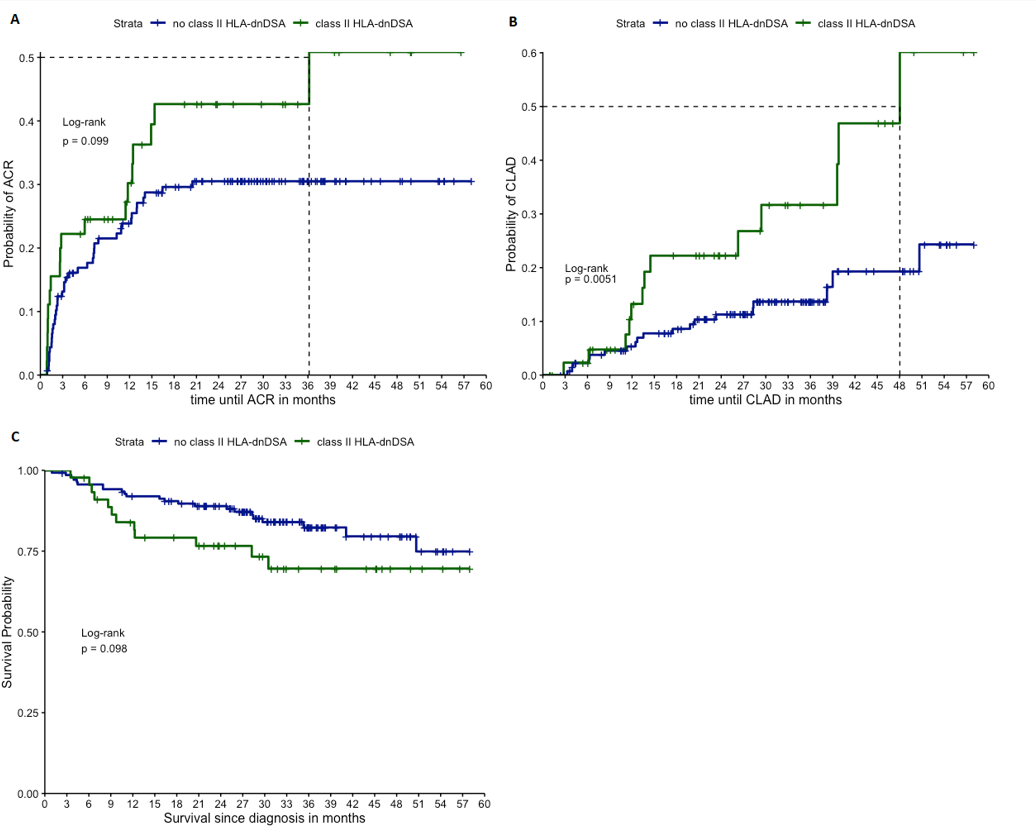


Notes: Kaplan-Meier curves of time until ACR, time until CLAD and time until death, stratified by development of class II HLA-dnDSA. P-values from LogRank test.

ACR = acute cellular rejection, CLAD = chronic allograft dysfunction, HLA-DQ-dnDSA = de-novo donor-specific antibodies against HLA-DQ locus

**Supp. Mat. 5 – Capsule sentence abstract**

Specific eplet- and antigen mismatches in lung transplant patients can lead to a higher risk of developing de-novo donor-specific HLA-DQ antibodies, which can impact clinical outcomes such as antibody-mediated rejection, acute cellular rejection, chronic lung allograft dysfunction

**Supp. Mat. 6 – Graphical abstract**


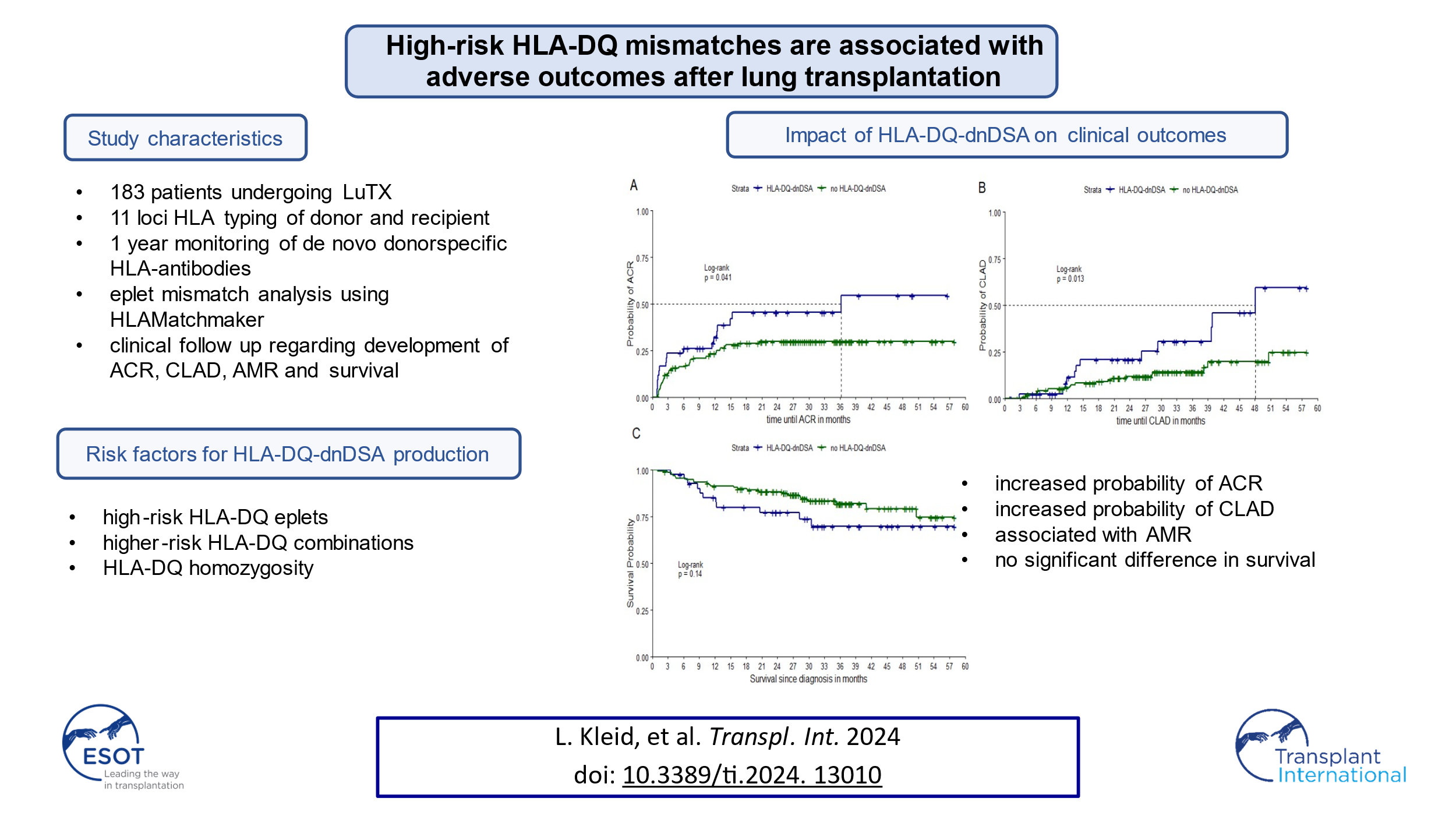

Supplement: Supplementary file 1 [file DataSheet1.docx]
